# Patient-reported symptoms at follow-up after a ureteral colic attack as an indication of spontaneous stone passage/remaining ureteral stone or presence of urinary tract obstruction

**DOI:** 10.1177/02841851251393798

**Published:** 2025-11-25

**Authors:** Klara Sahlén, Pär Dahlman, Per Liss, Maria Lönnemark, Monica Segelsjö, Anders Magnusson

**Affiliations:** Department of Surgical Sciences, Section of Radiology, 59561 Uppsala University Hospital, Uppsala, Sweden

**Keywords:** Urinary, CT, adult, calculi, symptoms, obstruction

## Abstract

**Background:**

Patient-reported symptoms is considered a possible alternative to computed tomography (CT) for the evaluation of remaining ureteral stones at follow-up after a ureteral colic attack.

**Purpose:**

To investigate patient-reported symptoms at follow-up after a ureteral colic attack in patients with a remaining ureteral stone and to assess the association of symptoms with degree of urinary obstruction.

**Material and Methods:**

This was a prospective study of 81 patients (68 men, 13 women; mean age = 59 years; age range = 23–88 years) referred for a follow-up CT after a ureteral colic attack with a remaining unilateral ureteral stone. A dynamic enhanced scan defined urinary obstruction. Stone size was the largest diameter of three multiplanar reformatations (axial, coronal, sagittal). Stone location was registered as proximal, mid-ureter or distal. Symptoms were registered in standardized questionnaires by the patient at the time of the follow-up.

**Results:**

In total, 43/81 (53%) patients reported symptoms. Obstruction of any degree was present in 16 (20%) patients. In 7 (100%) patients with moderate-severe obstruction reported discomfort attributed to the stone. In patients with mild obstruction, 5 (56%) reported discomfort; in patients with no obstruction, 31 (48%) reported discomfort.

**Conclusion:**

Almost half of the patients at follow-up reported no discomfort despite having a stone in the ureter. A significant association was found between a higher degree of obstruction and reported symptoms. Asymptomatic stones and silent partial obstruction could be missed based on reported symptoms. Imaging is still required to evaluate stone passage at follow-up after a ureteral colic attack.

## Introduction

Ureterolithiasis is a common disease with an increasing incidence worldwide. The condition is associated with morbidity and mortality and increasing cost to healthcare systems (1). In Sweden, the incidence of an acute ureteral colic attack is in the range of 1.4–1.8/1000 inhabitants and year (2). An acute ureteral colic attack caused by a ureteral stone typically presents with sudden onset of intermittent flank pain, which may move to the groin as the ureteral stone moves along the course of the ureter.

A clinically suspected ureteral colic is primarily treated with non-steroidal anti-inflammatory drugs, such as diclofenac, and if the diagnosis is clear, pain is tolerable, and no infection is present, watchful waiting is considered (3) since most ureteral stones are expected to pass spontaneously (4). If the patient is not pain-relieved, fever is present, or there is a suspicion of alternative diagnoses, an acute computed tomography (CT) examination of the urinary tract is performed (2). As is routine in Sweden, all patients with a suspected or verified ureteral stone are referred for a follow-up CT scan 2–3 weeks after the ureteral colic attack (5).

This routine is due to the risk of renal parenchymal damage and loss of renal function caused by urinary obstruction (6,7). The exact length or degree of obstruction that will result in renal damage is not known and is probably influenced by if the obstruction is partial or complete and by co-morbidity in the patient. However, studies on this correlation are difficult to perform in a human population and have experimentally been observed in animal studies.

An alternative to a routine CT follow-up could be to inquire for symptoms in patients and only follow-up radiologically if they are reported by the patient. This strategy has been considered a potential option for follow-up of distal stones <4 mm in size in the latest Swedish national guidelines for the treatment of upper urinary tract stones (5). However, there is limited evidence of the accuracy of patient-reported symptoms as an indication of remaining ureteral stones, and the presence of asymptomatic ureteral stones have also been identified (6). It would be of interest to assess the patient-reported symptoms at follow-up and evaluate their association to urinary tract obstruction, which is a risk factor to develop renal failure on the stone-bearing side (7).

The aim of the present study was to investigate the relationship between the patient's subjective discomfort attributed to the ureteral stone and degree of outflow obstruction at follow-up after a ureteral colic attack.

## Material and Methods

### Study population

**Fig. 1.**
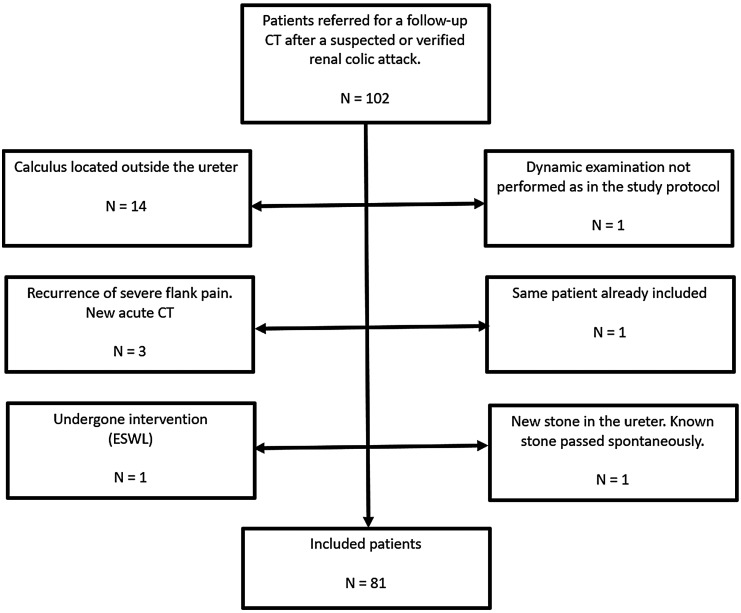
Flow chart of exclusion criteria.

This was a prospective study conducted at Uppsala University Hospital, Sweden between December 2016 and December 2022. Ethical approval was given by the regional ethical review board in Uppsala (reference no. 2016/473). A total of 102 patients were included in the study. In total, 21 patients were excluded (the exclusion criteria are presented in Fig. 1), leaving 81 patients (68 men, 13 women; mean age = 59 years; age range = 23–88 years) finally included in the study. The inclusion criteria were as follows: age ≥18 years; capable of completing a questionnaire in Swedish; referred for a follow-up CT scan after a suspected or verified ureteral colic attack; and in whom a remaining unilateral ureteral stone was detected. Further, the patient should not have undergone any form of intervention (such as shockwave lithotripsy or surgery) since the ureteral colic attack.

### CT settings

The CT scanners used were Somatom Definition Edge and Somatom Definition Flash (Siemens Medical Solutions, Forchheim, Germany), with collimation of 128 × 0.6 mm. The non-enhanced scan was performed with an automatic tube current modulation CARE Dose 4D, with the following parameters: quality reference = 60 mAs; Care kV reference = 120 kV; rotation time = 0.5 s; and pitch = 0.6. Image reconstruction was done with slice thickness and increments of 3/2.5 mm in the axial plane and 5/5 mm in the coronal plane. The contrast-enhanced dynamic scan was performed using the CARE Dose with the following parameters: 70 mAs and 100 kV (acquisition 1 × 10 mm); and rotation time = 0.36 s during the first minute, scans at 1–5 min were performed with 40 mAs.

### Follow-up ct-protocol

Before the examination, patients were instructed to drink 1000 mL of water and not to void 2 h before the examination. The follow-up protocol consisted of an initial non-enhanced scan of the entire urinary tract, which was evaluated by a radiologist while the patient was still in the CT scanner. If a ureteral stone was detected, a dynamic scan was performed. The dynamic protocol has been described in detail in a previous study (8). After contrast administration, repeated stationary scans visualizing the renal pelvis were conducted at given time points. Depending on the patient’s body size, 30– 40 mL of Iohexol 350 mg I/mL (Omnipaque; GE Healthcare, Oslo, Norway) or Iomeprol 400 mg I/mL (Iomeron; Bracco Imaging Spa, Milan, Italy) was administered at a rate of 4 mL/s with a power injector (Stellant D; Medrad, Indianola, PA, USA). This was followed immediately by a 50 mL saline bolus injection at the same rate. The dynamic scan started automatically with a delay of 20 s after contrast injection and was repeated every 10 s up to 60 s and then every 30 s until bilateral excretion was detected or until 300 s had passed. If no excretion was seen in the affected kidney in the first 300 s, another scan was performed after 30 min. When needed, a selective scan from 5 cm above to 5 cm below the stone was performed in a late phase to confirm the calculus position in the ureter (to distinguish it from a phlebolith).

### Questionnaire

Each patient filled in a standardized questionnaire after the CT examination regarding their symptoms at follow-up. Two questions were relevant for the objective of this study. The first question was whether the patient had any symptoms, and to that the patient could answer “yes” or “no.” The second question was to specify what type of discomfort they were experiencing if they had answered “yes” to the first question. Three common types of discomfort associated with ureteral stones were chosen as pre-existing options (“pain,” “dull pain,” and “urge to void”), as well as an opportunity for the patients to describe other types of symptoms (“other”). The questionnaire was evaluated for any issues on the first 10 patients.

### Grading urinary obstruction

Degree of urinary obstruction was defined by time taken for contrast medium to be excreted to the renal pelvises in the dynamic scan. Symmetrical excretion of contrast medium was graded as follows: 0 = no urinary obstruction (excretion observed within 5 min); 1 = mild obstruction (a difference in excretion time of 60 s within 5 min); 2 = moderate obstruction (excretion on the affected side observed at 5–30 min); and 3 = severe obstruction (no excretion on the affected side seen after 30 min). The grading was done by two observers (AM, an experienced uroradiologist, and KS, a third-year radiology resident) who were blinded to the results of the questionnaire.

### Stone characteristics

All stones were measured by two observers (AM and KS) in three multiplanar reformations (axial, coronal, and sagittal) in a soft-tissue window (WC 50, WW 400). The largest diameter in any of these planes was registered. The location of the stone in the ureter was defined as proximal if located above the sacroiliac joint, mid-ureter if located overlying the sacroiliac joint, and distal if located below.

### Statistical analysis

Qualitative variables are presented as counts with associated percentages, continuous variables with mean values with range. Fisher’s exact test was used to evaluate the relationship between symptoms and degree of obstruction. Due to the low number of patients in each category of obstruction, the moderate and severe degrees were combined into one group.

## Results

### Degree of obstruction and stone characteristics

A total of 65 (80%) patients did not have any degree of obstruction. Urinary obstruction was present in 16 (20%) patients. Mild obstruction was present in nine patients, moderate obstruction in five patients, and severe obstruction in two patients. The stones were located in the right ureter in 40 patients and in the left ureter in 41 patients. Mean stone size was 7 ± 3 mm (range = 3–14 mm). Stones were located in the proximal ureter in 25 patients, mid-ureter in seven patients, and located in the distal part of the ureter in 49 patients. Stone characteristics, location, and degree of obstruction are presented in Table 1.

**Table 1.** Stone size, location, and degree of obstruction.

Degree of obstruction	No. of patients (n = 81)	Proximal (n = 25)	Mid-ureter (n = 7)	Distal (n = 49)	Stone size (mm)
0	65	16 (25)	5 (8)	44 (68)	7.0 ± 3.0 (3–14)
1	9	4 (45)	2 (22)	3 (33)	8.0 ± 3.0 (4–11)
2	5	3 (60)	0 (0)	2 (40)	9.0 ± 3.0 (5–13)
3	2	2 (100)	0 (0)	0 (0)	7.0 ± 0.4 (7–7)

Values are given as n (%) or mean ± SD (range).

### Symptoms

The detailed results of reported symptoms are presented in Table 2. The presence of symptoms at follow-up was reported as “yes” or “no.” Of the 81 patients, 43 (53%) reported that they had symptoms and 38 (47%) reported that they did not. Symptoms were specified into the categories “pain,” “dull pain,” “urge to void,” and “other.”

**Table 2.** Symptoms reported at follow-up.

Symptoms	No. of patients
No symptoms	38
Pain (only)	2
Dull pain (only)	18
Urge to void (only)	8
Pain and dull pain	3
Pain and urge to void	6
Pain and “other”	0
Dull pain and urge to void	3
Dull pain and “other” (burning micturition n = 1, hematuria n = 1)	2
Urge to void and “other”	0
Pain and dull pain and urge to void	1
Total	81

### Symptoms and degree of obstruction

**Fig. 2.**
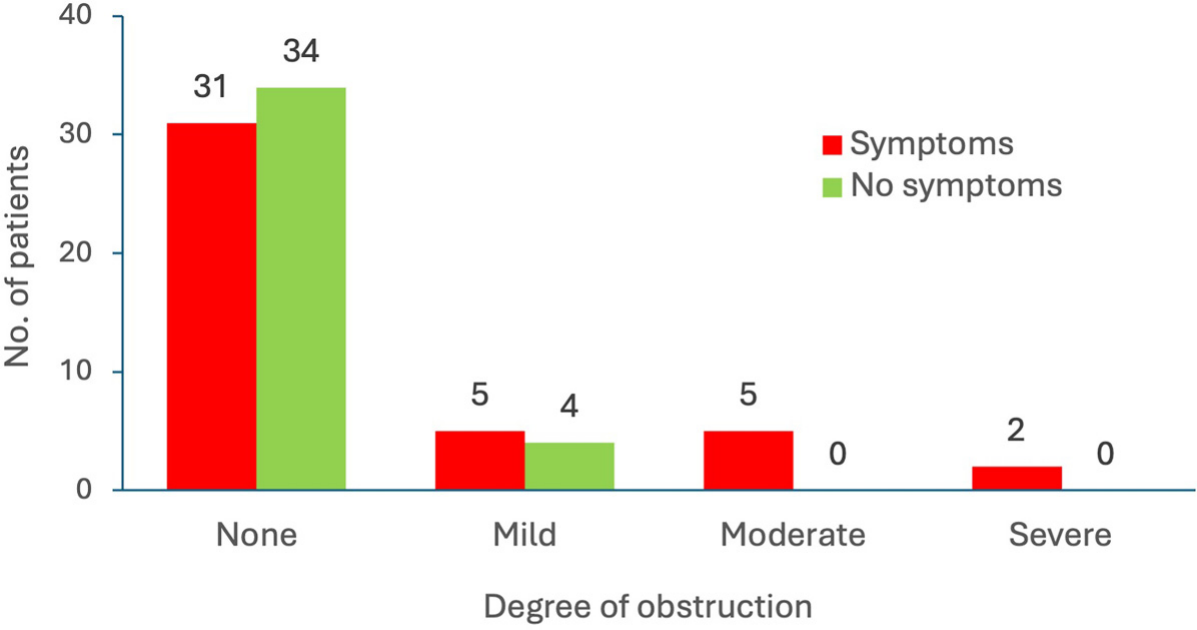
Degree of obstruction and symptoms (yes/no).

Degree of obstruction and presence of symptoms are presented in Fig. 2. There was an association between degree of obstruction and presence of reported symptoms at follow-up as “yes” or “no” (*P* = 0.029). Regarding specific symptoms, an association between degree of obstruction and pain (*P* = 0.048) was found, while no association was observed for dull pain (*P* = 0.34) or urge to void (*P* = 0.082).

## Discussion

At the follow-up after a ureteral colic attack, 43 (53%) patients reported symptoms attributed to the stone whereas 38 (47%) did not, despite having a stone in the ureter. Similar findings of cessation of pain but unsuccessful spontaneous passage were reported by Pandey et al. (40%) (9) and McLarty et al. (45%) (10). In comparison, Hernandez et al. (11) investigated asymptomatic patients at follow-up of which 26% still had a remaining ureteral stone; in a study by Jackman et al. (12), 84% of patients with a persisting stone at follow-up reported no symptoms.

Urinary obstruction of any degree was present in 16 (20%) patients in the study population, which is in line with a previous study on follow-up after a ureteral colic attack (8) where 28% had any degree of obstruction. All patients with moderate to severe obstruction (n = 7) reported symptoms at follow-up. In the group with mild obstruction, 56% reported symptoms; in the group with no obstruction, 49% did. Subsequently, approximately 44% and 51% of the patients in these groups, respectively, reported no symptoms. A significant association between a higher degree of obstruction and symptoms reported as “yes” or “no” (*P* = 0.029) and reported pain (*P* = 0.048) was found. This finding is in line with the expectation of symptoms associated with urinary obstruction by a ureteral stone. No other similar study assessing symptoms and obstruction was found for comparison. To our knowledge, this is the first study to evaluate urinary obstruction by contrast medium excretion and presence of symptoms at follow-up.

The results indicate that patient-reported symptoms as a screening tool for remaining ureteral stone after a ureteral colic attack is an uncertain follow-up method as almost half of the patients were asymptomatic even though they had a ureteral stone present at follow-up.

All patients with moderate-severe obstruction would be included for follow-up but a few asymptomatic patients with mild obstruction would not be included. Thus, silent partial obstruction was detected in some of the patients.

There could be an increased risk of renal function impairment if urinary obstruction is prolonged (13); however, the exact extent to which a kidney can endure complete or partial obstruction is not known. As the CT scan only gives a snapshot of the state of the patient, and given that stones may alter location and impaction over time, it must be considered that degree of obstruction also may change. An increased degree of obstruction would be expected to be symptomatic, and this was supported by the findings of this study, yet silent obstruction remains a diagnostic problem.

The mean stone size was 7 ± 3 mm (range = 3–14 mm). Only 13 (16%) stones were ≤4 mm in size (two were 3 mm and 11 were 4 mm), probably reflecting that smaller ureteral stones have a higher probability of spontaneous passage. A total of 23 (30%) stones measured ≤5 mm. The mean size and proportion of stones ≤5 mm at follow-up was similar to that reported by McLarty et al. (7.6 mm, 25%) (10).

A strength of this study was the use of a quantitative and standardized method to evaluate the presence and degree of obstruction. The population size is a limiting factor, especially for the evaluation of stones causing obstruction as obstruction was scarce. Because a large proportion of ureteral stones will pass spontaneously, remaining stones are quite scarce at follow-up and despite a consecutive inclusion of patients for almost 6 years at our department, the study population is relatively small. A multicenter study may be required to yield a larger study population. Symptoms are subjective and may have different meanings to different individuals, which is a limitation to questionnaires but reflects what daily praxis would be for the urologist who evaluates the patient. The questionnaire was designed to find as many symptomatic patients as possible; however, the panorama of symptoms attributed to a ureteral stone is wide and other typical symptoms could have been chosen as predefined options. Considering the relatively large mean stone size, the results are not applicable on smaller stones which may have a different relationship to presence of symptoms and obstruction at follow-up.

The dynamic scan is a novel approach to grading urinary tract obstruction. The normal time to excretion in kidneys is estimated at approximately 3 min (8) and the other kidney can be considered the normal reference in the individual patient. Through a dynamic series, it is possible to grade urinary tract obstruction objectively. A grading of the severity of obstruction may help the clinicians in management decision.

The radiation dose associated with the study protocol was evaluated in a previous study (8). The mean effective dose for a patient was 2.4 mSv (non-enhanced and dynamic scan), which was 1.8 mSv (43%) less than the dose in a traditional follow-up protocol (non-enhanced and excretion phase [4.2 mSv]). The added radiation dose from the dynamic series was 0.3 mSv. After completion of the study, it was decided to reduce the number of scans in the dynamic protocol to a maximum of four (at 3 min, 4 min, 5 min, and 30 min, or end when bilateral excretion is observed) to further reduce the dose to the patient. The radiation dose from a CT scan of the urinary tract is continuously decreasing with ALARA in mind. Ultra-low-dose protocols and photon CT as well as ultrasound are promising alternatives to the low-dose in the future.

In conclusion, almost half of the patients at follow-up reported no discomfort despite having a stone in the ureter. A significant association was found between a higher degree of obstruction and reported symptoms. Asymptomatic stones and silent partial obstruction could be missed based on reported symptoms. Imaging is still required to evaluate stone passage at follow-up after a ureteral colic attack.
